# Mass occurrence of seep-specific bivalves in the oldest-known cold seep metazoan community

**DOI:** 10.1038/s41598-017-14732-y

**Published:** 2017-10-30

**Authors:** Michal Jakubowicz, Krzysztof Hryniewicz, Zdzislaw Belka

**Affiliations:** 1 Institute of Geoecology and Geoinformation, Adam Mickiewicz University, ul. B. Krygowskiego 10, 61-680 Poznań, Poland; 2 Institute of Paleobiology, Polish Academy of Sciences, ul. Twarda 51/55, 00-818 Warszawa, Poland; 3 Isotope Laboratory, Adam Mickiewicz University, ul. B. Krygowskiego 10, 61-680 Poznań, Poland

## Abstract

One of the most striking features of modern chemosynthesis-based ecosystems surrounding methane seeps is the presence of abundant chemosymbiotic bivalves. However, such accumulations have rarely been reported from Palaeozoic to mid-Mesozoic seeps, and it is widely thought that general trends in the evolution of chemosynthetic communities paralleled those typifying most marine environments, with the bivalve prevalence starting in the Mesozoic and with Palaeozoic seeps being dominated by brachiopods. Here, we report a discovery of bivalve clusters in the oldest-known methane seep that hosted metazoan fauna, dated to the late Silurian. We identify the bivalves, externally very similar to modern chemosymbiotic forms, as members of the extinct family Modiomorphidae, known previously from a younger, Devonian seep. The bivalves inhabited the seep at a stage of increased fluid flow, when they co-occurred with atrypid brachiopods, and display a set of morphological characteristics suggesting a seep-obligate lifestyle. We conclude that bivalves colonised chemosynthesis-based ecosystems at least as early as brachiopods and apparently first developed specialized lineages able to thrive in seep-related habitats for a prolonged period of time. Rather than being simple ecological successors of brachiopods, rich bivalve communities represent an ancient and recurring theme in the evolution of chemosynthetic assemblages.

## Introduction

Modern ecosystems based on chemical energy sources supplied by methane (cold) seeps and hydrothermal vents stand out as some of the most unique communities found in the deep sea^1–4^. These both nutrient- and toxin-rich settings host prolific, highly-endemic faunas, the most characteristic elements of which include vestimentiferan tube worms and mass concentrations of large bivalves, notably bathymodiolin mussels and vesicomyid clams^3,5,6^. Having developed close symbioses with chemoautotrophic bacteria harboured in their gills, the bathymodiolins and vesicomyids have dominated many seeps and vents since the mid-Palaeogene, the moment often regarded as the onset of modern-type chemosynthetic ecosystems^2,7–10^.

Despite three decades of studies that have aimed to better constrain the fossil record of chemosynthesis-based assemblages, the early stages of their evolution remain poorly recognised. As few as six metazoan methane seep ecosystems have been documented for the entire Palaeozoic^2,11^, and these were often inhabited by biota with no or unclear affinities to modern seep lineages^2,8,12^. As a result, many key questions regarding the palaeoecology of Palaeozoic seeps remain unanswered, and few attempts have been made so far to delineate general trends in the evolution of the earliest chemosynthetic communities. Probably the most widely held perception has become that, unlike modern, bivalve-dominated seeps, the Palaeozoic to late Mesozoic seep ecosystems were dominated by brachiopods^7,13^. The role of bivalves at seeps until the early Mesozoic was poorly known and considered subordinate, with the notable, yet apparently isolated exception of a single Devonian seep that sustained dense bivalve accumulations, but few, lingulate brachiopods^12,14,15^. Likewise, although the Devonian seep bivalves apparently possessed specialised features indicative of their longer evolution at seeps^12^, no molluscs were known from the sole example of an older, Silurian, metazoan-containing seep ecosystem^16^. Nevertheless, the available Palaeozoic record appears too fragmentary to support such broad generalisations. Indeed, even our recognition of the documented Palaeozoic seeps is often very limited and turns out, in some cases, strikingly incomplete.

The latter situation is exemplified by the present study, in which we report the presence of mass accumulations of large bivalves at the oldest-known, Silurian methane seep, an occurrence that remained unnoticed despite several previous studies^16–18^. The seep deposit, found near El Borj in Moroccan Meseta, has so far been documented to contain dense clusters of the atrypid brachiopod *Septatrypa lantenoisi* ^17^, putative remains of microbial structures^16^, and rare fossils of possibly tube-worm origin^18^. In addition to discussing the taxonomic affinities and palaeoecology of the bivalves, we also resolve a major interpretational controversy regarding the exact composition of the seeping fluids^11,18^, providing evidence that the El Borj carbonates formed at a classical, hydrocarbon-issuing seep. The study shows that bivalves with considerably specialised morphology were present at seeps at least 420 Ma, thus preceding the oldest record of the supposedly most seep-specialised lineage of brachiopods by ~50 myr^19^.

## The El Borj seep site

The El Borj deposit comprises a carbonate body several tens of m wide and ~15 m thick, embedded within predominantly argillaceous, shaly sediments^16,18^. The adjacent strata represent a mélange of rocks, ranging from upper Silurian to Carboniferous in age, a sequence also containing other seep deposits in the Moroccan Meseta^19^ and indicative of gravitational slumping and deformation of the El Borj body within a Carboniferous basin (see Supplementary Discussion). The age of the limestones remained, until now, poorly constrained and tentatively assigned to the uppermost Silurian^17,18^. The conodont data collected in the course of the present study date the formation of the seep to the late Ludfordian, making it thus somewhat older than previous assumptions^18^ (see Supplementary Discussion and Supplementary Fig. 1).

**Figure 1:**
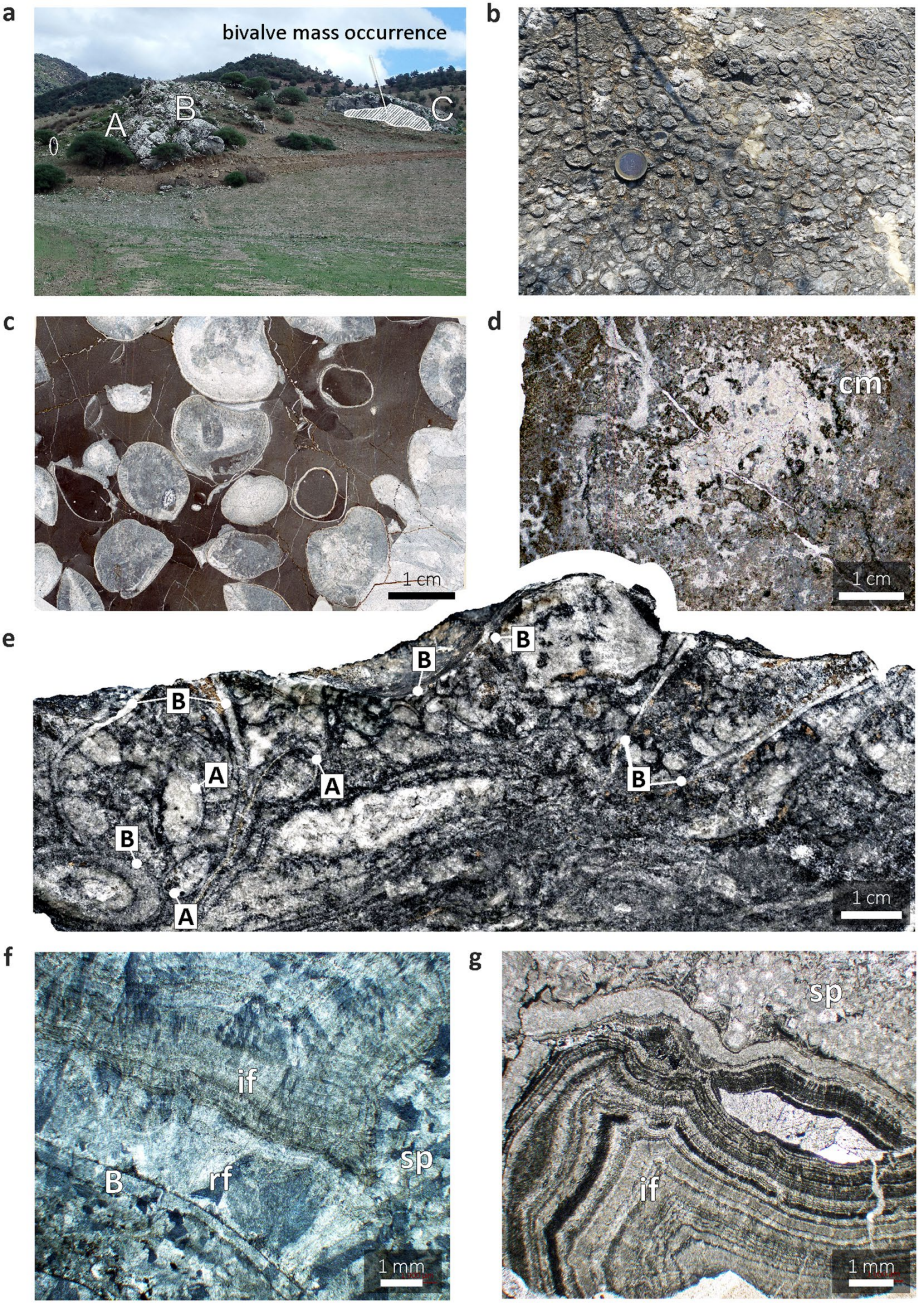
Methane seep carbonates of El Borj. (**a**) Overview of the El Borj deposit with indicated constituent facies (units A–C; see text) and area where the bivalve clusters occur (shaded). Man (1.85 m, encircled) for scale. (**b,c**) Clusters of the atrypid brachiopod *Septatrypa lantenoisi* (unit B) observed in field (**b**; coin, 23 mm in diameter, for scale) and thin-section (**c**) views. (**d–g**) Seep carbonates hosting the assemblage of *S. lantenoisi* and the modiomorphid *Ataviaconcha* bivalves (unit C). (**d**) Irregular, micropeloidal fabric typical of the seep carbonates resulting from the abundance of microbial-derived, micritic clots (cm). (**e**) Polished slab showing a typical, intricate appearance of the seep carbonates enclosing the modiomorphid bivalves (B) and atrypid brachiopods (A). (**f,g**) Two textural varieties of laminated early cements engulfing the bivalve and brachiopod shells: radiaxial-fibrous calcite (rf; partially recrystallised to a sparry mosaic, sp) and isopachous fibrous calcite (if). Note irregular dissolution surfaces separating some of the laminae (indicated by the arrow in (**g**).

**Figure 2:**
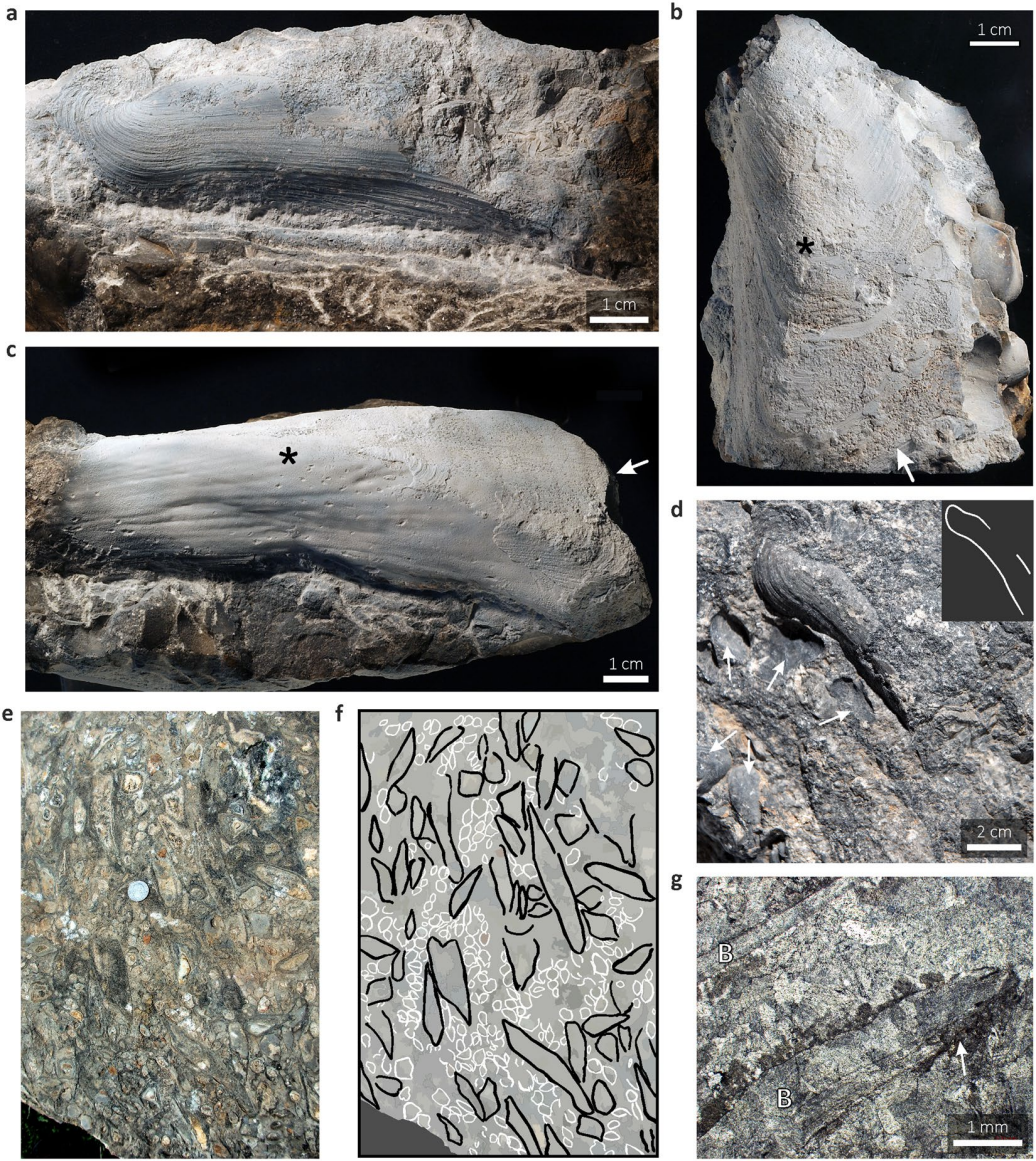
The modiomorphid bivalve *Ataviaconcha* sp. from El Borj. (**a**) Left-lateral view of a left valve, with partially preserved anterior lobe. (**b**) Left-lateral view of a partially preserved left valve showing well developed posterior lobe (arrow); note strong carina extending from the anterior towards the posterior lobe (asterisk). (**c**) Left-lateral view of a partially preserved internal mould of a left valve; note an enlarged and fan-shaped posterior lobe (arrow) and carina extending from the anterior towards the posterior lobe (asterisk). (**d**) Field view of a large, incurved *Ataviaconcha* specimen (outline shown in the inset). The few visible specimens of the brachiopod *Septatrypa lantenoisi* are indicated with arrows. (**e,f**,) Field view (**e**) and corresponding schematic drawing (**f**) of the bivalve-brachiopod assemblage (black – bivalves; white – brachiopods). Note the common deformations of the bivalve shells due to their diagenetic recrystallisation and partial dissolution. Coin (**e**); 21 mm in diameter) for scale. (**g**) Thin-section view (cross-polarised light) of two bivalve shells (B). Note recrystallisation of the original shell material to a sparry calcite mosaic, with preservations of some remnants of the original multi-layered structure. The shell was partially corroded and coated with clotted micritic carbonate (arrow).

The carbonates reveal a range of petrological features typical of limestones precipitating due to anaerobic oxidation of methane^16,18^ (Fig. 1; Supplementary Figs 1 and 2). The deposit encompasses a succession of three petrographically distinct units, termed Units A–C by Barbieri et al.^16^ (Fig. 1a): Unit A, composed of hematite-rich alternations of limestones and marls; Unit B, made up of massive micritic carbonates with abundant brachiopods and scarce sparry cements (Fig. 1b,c); and Unit C, consisting chiefly of multiple generations of carbonate cements. The occurrence of the bivalves is limited to Unit C (Fig. 1a), in which they co-occur with brachiopods, represented by the same sulcate, thin-walled, smooth-shelled atrypid identified as *S. lantenoisi* ^17,18^ that dominates the underlying micritic carbonates. The paragenetic sequence here is very complex (Fig. 1d–g); volumetrically dominant are early isopachous cements that form irregular rims on all available surfaces (Fig. 1e–g). Despite clear diagenetic recrystallisation, the isopachous cements preserve well-defined traces of the original fibrous fabrics (Fig. 1f–g). Clotted textures and disseminated pyrite crystals are locally common (Fig. 1d). Growth of the cements was periodically interrupted by episodes of corrosion marked by dissolution surfaces (Figs. 1g and 2g).

## The oldest seep-related bivalves

**Figure 3:**
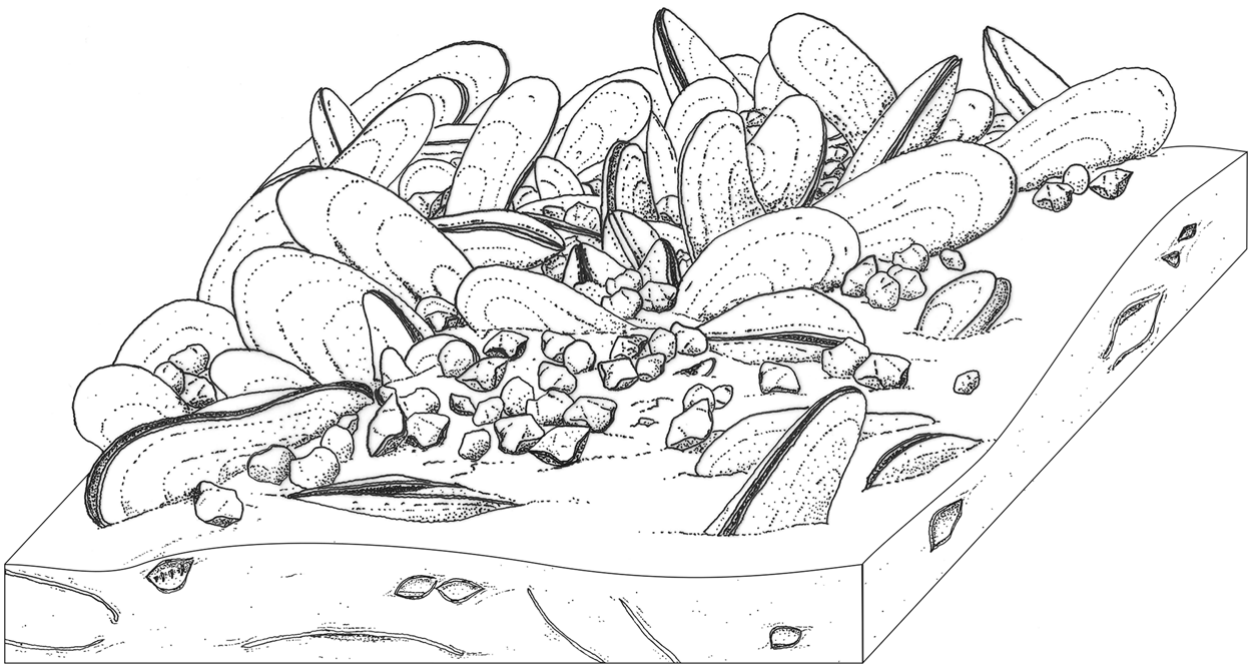
Schematic reconstruction of the assemblage of modiomorphid bivalves and atrypid brachiopods that inhabited the Silurian methane seep of El Borj.

The bivalve clusters reach densities of up to 250 individuals/m^2^; many specimens display consistent orientations (Fig. 2e,f), presumably reflecting their original alignment. Owing to their small sizes (max. 32 mm wide), the co-occurring brachiopods form dense aggregates among the bivalves (Figs. 2d–f and 3; Supplementary Fig. 1). Most of the bivalve shells were pervasively recrystallised or dissolved during diagenesis, which resulted in frequent deformations of their original outlines (Fig. 2e,f). Nonetheless, in some cases undeformed specimens can still be observed, enabling genus-level taxonomic recognition. The bivalves are large (up to 160 mm long), with highly elongated, fan-shaped posterior shell lobe (Fig. 2a–c). The ventral shell margin is indented by a broad sinus, giving the shells a characteristic, boomerang-like shape (Fig. 2d). This feature, together with the shape of the anterior lobe (Fig. 2a), strong carina (Fig. 2b–c), and the pattern of the muscular and ligament attachments, places the El Borj bivalves in the genus *Ataviaconcha*, known so far only from a Middle Devonian seep in the eastern Anti-Atlas, where it also forms mass concentrations^12^. *Ataviaconcha* belongs to the extinct family Modiomorphidae, a group morphologically convergent with, but evolutionarily distant from, extant mussels^6,10,20^. Except for the two Moroccan assemblages of *Ataviaconcha*, reports of Palaeozoic seep-related bivalves are limited to scarce solemyids found at Middle Devonian^12^ and lower Carboniferous seeps^21^. In addition, a putative modiomorphid has been documented from a Silurian hydrothermal vent^22^. However, none of these bivalves has been reported to form dense accumulations around Palaeozoic fluid emissions.

The peculiar morphological characteristics of *Ataviaconcha* from El Borj, mostly shared with their Devonian congener^12^, most likely represent advanced adaptations to a semi-infaunal lifestyle in a seep-related habitat. Similar elongated, variously incurved shells developed independently in several groups of semi-infaunal seep bivalves, including chemosymbiotic vesicomyids and bathymodiolins^5,6,23^, as well as another, Mesozoic lineage of modiomorphoid bivalves^10,24,25^. Such shells, when oriented with their anterior end shallowly buried in the sediment, and the posterior part exposed, enable simultaneous access to seawater-derived oxygen and interstitial sulphide^3,12,23^. Given the high metabolic toxicity of sulphide, the semi-infaunal strategy provides no obvious advantage to non-chemosymbiotic bivalves^5^. Thus, the elongated shell of *Ataviaconcha* strongly suggests close reliance on reduced compounds and oxic seawater, a physiological trait exhibited by bivalves living in symbiosis with chemoautotrophic bacteria. This is further supported by the large size of the shell, a common distinctive feature of chemosymbiotic molluscs^3,6^, which places *Ataviaconcha* among the largest Palaeozoic bivalves known to date. The bivalve gills appear generally well suited to acquire chemosymbionts, with no advanced morphological adaptations required, as shown by the independent development of chemosymbiosis in several groups of Bivalvia, including Solemyidae, one of the most basal bivalve groups^6,20^. Since a chemosymbiotic lifestyle was likely present in solemyid and lucinid bivalves as early as the Ordovician and the Silurian, respectively^6,20^, the seep-related modiomorphids may not, therefore, have been the most ancient bivalve lineage in which chemosymbiosis appeared.

## Habitat of the Silurian bivalve-brachiopod assemblage

**Figure 4:**
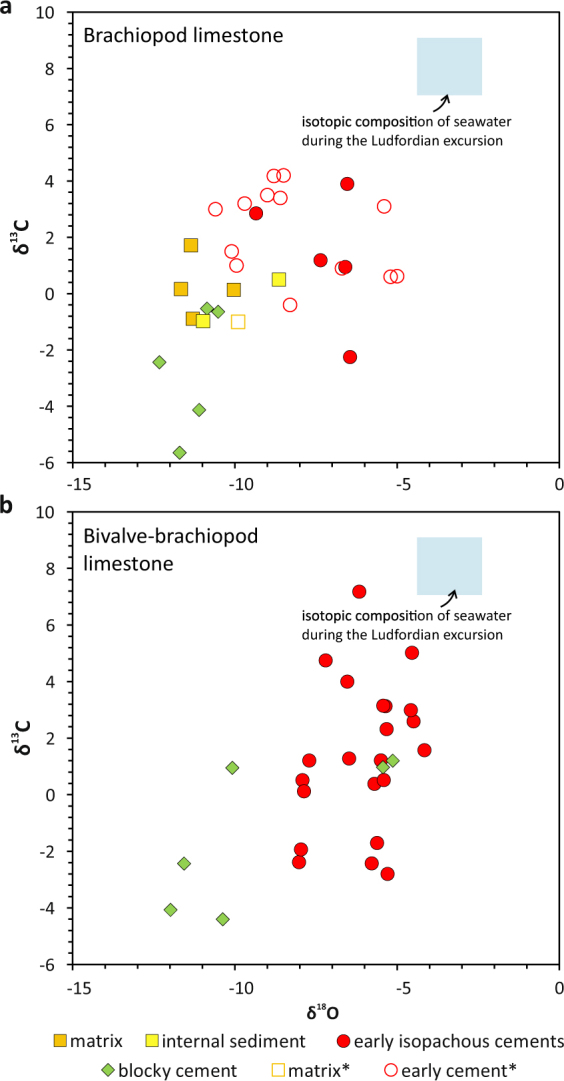
Carbon vs. oxygen isotope cross-plots for various carbonate phases found in the seep limestones hosting the monospecific brachiopod accumulation (Unit B; see text), and the bivalve-brachiopod assemblage (Unit C). The isotopic composition of contemporaneous ambient seawater (late Ludfordian isotope excursion)^26,27^ and data from an earlier study of Buggisch & Krumm^18^ (asterisks) are plotted for comparison. The error bars are smaller than the size of the symbols. All results are shown in ‰ V-PDB.

Compared to typical examples of seep carbonates, carbon isotope signatures of the El Borj limestones, ranging from −2.8 to +7.2‰ for the early diagenetic phases (Fig. 4 and Supplementary Table 1), appear anomalously heavy. At typical seeps, methane oxidation releases large quantities of isotopically light carbon, which results in strongly negative δ^13^C values in precipitating carbonates^2,15^. These signals have led to interpretations of the El Borj deposit as either strongly altered by diagenesis that overprinted originally more negative values^16^, or as having formed due to methane formation, rather than oxidation^11,18^. However, none of these scenarios offered a plausible explanation for the combination of palaeontological and geological features observed in the studied limestones (see Supplementary Discussion and Supplementary Fig. 3).

The unusual isotopic signals are, in turn, explained by our stratigraphic data. The late Ludfordian (late Silurian) age established by the conodont analyses places the formation of the El Borj deposit within a time interval characterized by a very prominent positive excursion in the carbon isotope composition of seawater^26,27^. This is critical for the interpretation of the isotopic signals, since, when corrected for the δ^13^C_seawater_ of +7 to +9‰ typical of the late Ludfordian excursion, the values measured in the early cements fall, in fact, down to 12‰ below the signatures of contemporaneous marine calcites. Although this^13^C depletion is still less significant than that characterising some modern seep carbonates^2^, such moderately negative ratios are typical of Palaeozoic seep limestones^15,18,19,21^. In the few instances where lower signals were measured, the seep carbonates contain few fossils, with both brachiopods and bivalves being notably absent^11,18^. Among other factors, it seems that many early seep-dwelling metazoans were less tolerant of the environmental toxicity of the most intense seeps, and preferred temperate emissions, at which lower hydrocarbon contents or diffuse flow resulted in less^13^C-depleted signatures of the carbonates. This appears particularly plausible for dimerelloid brachiopods, which are not known from contemporary seeps, and their presence at Palaeozoic and Mesozoic seeps is limited to carbonates characterized by moderate^13^C-depletions, with δ^13^C values typically ranging from several to −20‰, and only exceptionally exceeding −25‰^19,21,25,28–32^. While the El Borj site remains the only known seep inhabited by members of the order Atrypida, rather than Rhynchonellida and Terebratulida typical of younger, Late Devonian to Cretaceous seeps^33,34^, the preference for micrite-dominated, moderately^13^C-depleted seeps appears shared by all seep-related brachiopods.

Compared to the underlying, brachiopod-dominated facies, the bivalve-rich carbonates display more variable δ^13^C signals, and record both the lowest and highest values measured among the early cements (Fig. 4). Combined with the petrological observations, this can presumably be attributed to a transition from the stage of relatively slow seepage to a period of more vigorous, spatially and temporarily variable flow, which attracted the abundant seep-specialised bivalves. Since at modern seeps dense clusters of chemosymbiotic bivalves clear bottom waters of a significant proportion of toxic sulphide^5^, the appearance of the bivalves may have played an important role in enabling the continuous presence of the apparently less-specialised atrypids, as brachiopods are otherwise very rarely found in cement-dominated seep carbonates^28,29^.

## Bivalve vs. brachiopod dominance at seeps over time

**Figure 5:**
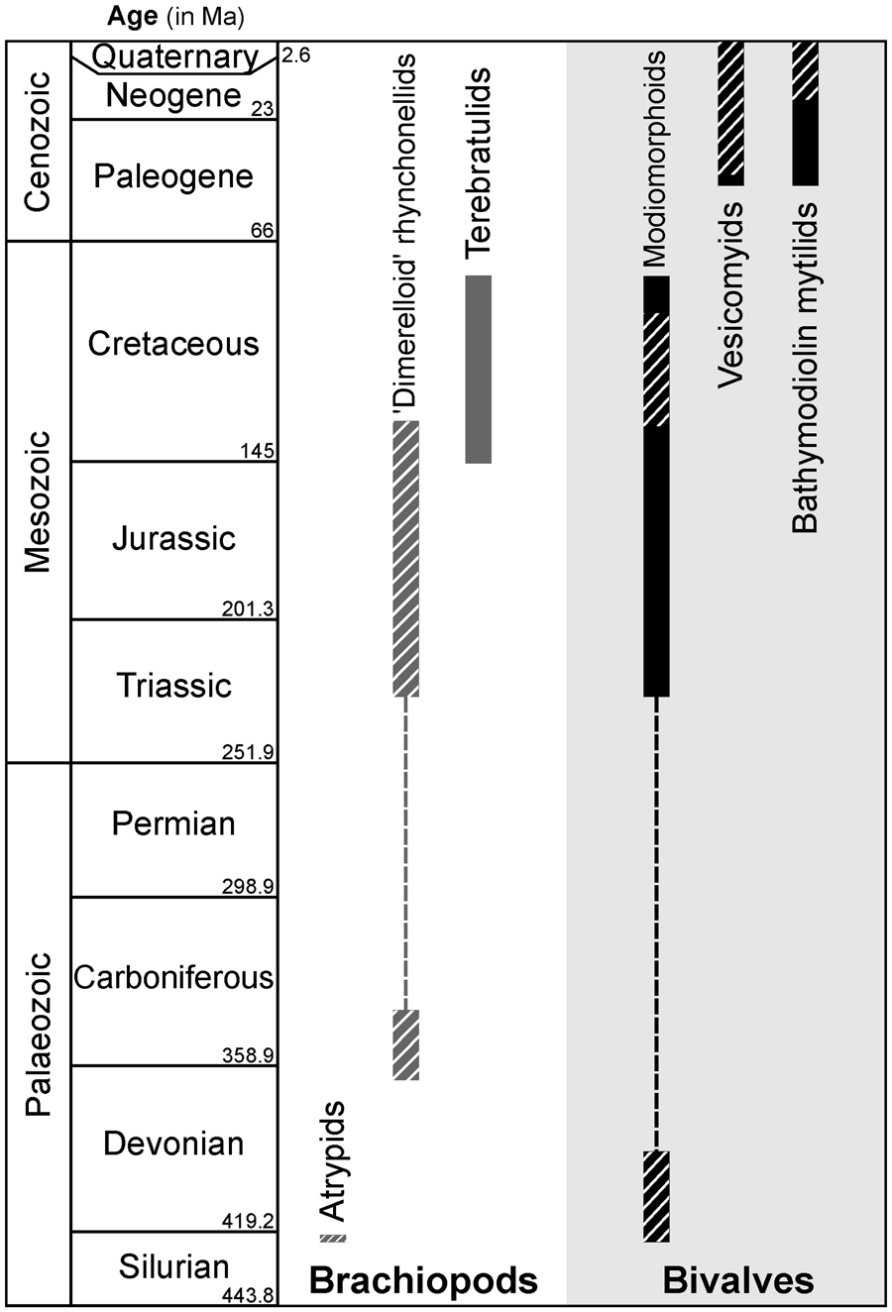
Stratigraphic ranges of different groups of articulate brachiopods^33,34^ and epifaunal to semi-infaunal bivalves^10,12^ in chemosynthesis-based communities throughout the Phanerozoic. Time periods for which representatives of different lineages are known to have formed mass concentrations at seeps are cross-hatched. Taxonomic relationships within the bivalve clade Modiomorphoida remain uncertain; in recent studies the Palaeozoic and Mesozoic seep-dwelling representatives of the group were placed in different families, Modiomorphidae and Kalenteridae, respectively^10,12^. The brachiopod superfamily Dimerelloidea is perceived here as including the three families of rhynchonellid brachiopods with abundant representatives at Palaeozoic and Mesozoic seeps: Halorellidae, Peregrinellidae and Dimerellidae^33,34,39^. The position of terebratulid brachiopods as seep-specialised inhabitants or opportunistic colonisers remains unclear^33,40^.

The present study confirms a previous suggestion that bivalves could have colonised seep-related ecosystems at least as early as brachiopods^12^. In fact, the *Ataviaconcha* modiomorphids remained present at seeps for at least 30 myr (Fig. 5) and reveal derived adaptations to reducing habitats^12^, whereas the abundant atrypids are known from an isolated occurrence. The former, therefore, can be perceived as more prominent inhabitants of the Middle Palaeozoic seeps.

The apparent disappearance of the bivalve-dominated seep ecosystems after the Middle Devonian is enigmatic, given that representatives of the modiomorphids, unlike the atrypid brachiopods, survived the Frasnian-Famennian extinctions. Despite the apparent physiological ‘inferiority’ often suggested for the Brachiopoda^35,36^, rich assemblages of dimerelloid brachiopods appeared at seeps in the Late Devonian, and were present in many of the late Palaeozoic to Early Cretaceous seep communities^30,33,34^. The next record of seep-related modiomorphoid bivalves occurs 170 myr later in the Late Triassic^28,31^. Subsequently, clusters of modiomorphoids re-appeared at seeps in the latest Jurassic and the Cretaceous^10^, but the relationships between the Palaeozoic and Mesozoic seep-related modiomorphoids remain dubious^10,12,24^. To some degree, the absence of rich seep bivalve assemblages from the Late Devonian to early Mesozoic may be attributed to the paucity of the fossil record as very few seeps have been reported from this period, with dense brachiopod clusters known from three of them^19,21,28^. In addition, the aragonitic shells of the bivalves are typified by much lower preservation potential than that of the low-Mg calcitic brachiopods, and, as illustrated by the present study, even at well-known seeps large and abundant, yet poorly preserved bivalves may long remain unnoticed.

The general scarcity of late Palaeozoic and early Mesozoic seeps could also have been of importance. It has been attributed to the continental configuration with restricted areas of continental margins that developed after the formation of the Pangaea supercontinent, and to the associated low levels of tectonic activity during that time^7,28^. In an ocean with rare, geographically distant seeps, a net result could have been limited advantage of advanced specialisation to seep-related habitats, creating favourable conditions for taxa applying more opportunistic strategies. The latter were probably more typical of the seep-related brachiopods, most likely devoid of chemosymbionts^12,34^. Indeed, the gradual decrease in the diversity of brachiopods in seep communities during the Jurassic and Cretaceous coincides approximately with the progressive Pangaea breakup, which was accompanied by the gradual restoration of the bivalve-dominated seep ecosystems^10,30,33^. Rather than being a typical pattern of pre-Cretaceous seep palaeoecology, the long period of the apparent brachiopod dominance at seeps may have, therefore, resulted from of a unique combination of geotectonic and palaeoenvironmental factors. As emphasised by the present study, not only in the late Mesozoic and Cenozoic, but also throughout a large portion of the middle Palaeozoic, dense clusters of large, seep-specialised bivalves could have, in turn, been a common form of chemosynthetic ecosystems. In terms of the dominant shelly fauna, contemporary seep assemblages represent a revival of a theme that first appeared in the evolution of chemosynthesis-based communities over 400 Ma.

## Methods

The palaeontological and petrological analyses have been conducted on both isolated and carbonate-embedded specimens of the modiomorphid bivalve *Ataviaconcha* sp. and atrypid brachiopod *Septatrypa lantenoisi*. The *S. lantenoisi* brachiopods are abundant in the micrite-dominated seep carbonates, from which they weather out easily, so that the analyses included several tens of isolated individuals. The bivalves, in turn, were typically firmly embedded within the carbonate cementstone, and the specimens were prepared in the laboratory using a hand-held vibrotool to the extent possible. Petrographic investigations were carried out on a few tens of large (7.5 × 5 cm) thin sections and polished slabs of the seep limestones. In addition to the plane- and cross-polarised, transmitted-light microscopic analyses, the thin sections were studied under cathodoluminescence (CL) with a Cambridge luminoscope system CITL 8200 mk3 (‘cold cathode’ type), operating under a 10–12 kV accelerating voltage and a 200–250 μA beam current.

Samples for isotopic measurements were collected from slabbed rock surfaces using a microscope-mounted microdrill. Thin sections corresponding to each slabbed surface were analysed prior to sampling to assist in the accuracy of drilling. Carbon and oxygen isotope measurements were performed on powdered carbonates at the Stable Isotope Laboratory of GeoZentrum Nordbayern (Friedrich-Alexander University of Erlangen-Nürnberg). CO_2_ was released from the carbonate phase at 70 °C using 103% H_3_PO_4_ with an automated Gasbench II sampling device, and analysed for carbon and oxygen isotopes with a Thermo-Fisher Delta V Plus mass spectrometer. All isotopic ratios are given in the standard δ notation, in ‰ relative to the V-PDB standard. Reproducibility of the measurements was monitored by analyses of laboratory standards calibrated to international standards NBS19 (δ^13^C = 1.95‰, δ^18^O = −2.20‰) and LSVEC (δ^13^C = −46.6‰, δ^18^O = −26.7‰). The average reproducibility (1σ) was ±0.07‰ for δ^13^C and ±0.06‰ for δ^18^O.

Strontium isotope analyses were conducted on carbonate powders in the Isotope Laboratory of the Adam Mickiewicz University in Poznań (Poland). Samples (~50 mg each) were dissolved at ~100 °C in closed PFA vials with 0.75 N HCl. Sr separation was carried out following a procedure developed by Pin *et al*.^37^ and Dopieralska^38^. Strontium was loaded with a TaCl_5_ activator on a single rhenium filament and measured for isotopic ratios in dynamic collection mode on a Finnigan MAT 261 multi-collector thermal ionization mass spectrometer. During the course of this study, the NBS 987 Sr standard was typified by a^87^Sr/^86^Sr ratio of 0.710230 ± 10 (2σ mean of twelve analyses). Total procedure blanks were <80 pg.

### Data availability

All data generated or analysed during this study are included in this published article (and its Supplementary Information files).

## Electronic supplementary material

Supplementary Information

## References

[1] Van Dover CL (2002). Evolution and Biogeography of Deep-Sea Vent and Seep Invertebrates. Science.

[2] Campbell K (2006). Hydrocarbon seep and hydrothermal vent paleoenvironments and paleontology: Past developments and future research directions. Palaeogeogr. Palaeocl..

[3] Dubilier N, Bergin C, Lott C (2008). Symbiotic diversity in marine animals: the art of harnessing chemosynthesis. Nat. Rev. Microbiol..

[4] Boetius A, Wenzhoefer F (2013). Seafloor oxygen consumption fuelled by methane from cold seeps. Nat. Geosci..

[5] Duperron, S. In *The Vent and Seep Biota: Aspects from Microbes to Ecosystems* Vol. 33 Topics in Geobiology (ed S. Kiel) 137–167 (Springer, 2010).

[6] Taylor, J. D. & Glover, E. A. In The *Ve*nt and *S*eep B*iota: Aspects from Microbes to Ecosystems* Vol. 33 Topics in Geobiology (ed S. Kiel) 107–135 (Springer, 2010).

[7] Campbell KA, Bottjer DJ (1995). Brachiopods and chemosymbiotic bivalves in Phanerozoic hydrothermal vent and cold seep environments. Geology.

[8] Little C, Vrijenhoek RC (2003). Are hydrothermal vent animals living fossils?. Trends Ecol. Evol..

[9] Kiel S, Little CT (2006). Cold-seep mollusks are older than the general marine mollusk fauna. Science.

[10] Jenkins R (2013). Worldwide distribution of the modiomorphid bivalve genus *Caspiconcha* in late Mesozoic hydrocarbon seeps. Acta Palaeontol. Pol..

[11] Himmler T, Freiwald A, Stollhofen H, Peckmann J (2008). Late Carboniferous hydrocarbon-seep carbonates from the glaciomarine Dwyka Group, southern Namibia. Palaeogeogr. Palaeocl..

[12] Hryniewicz K, Jakubowicz M, Belka Z, Dopieralska J, Kaim A (2017). New bivalves from a Middle Devonian methane seep in Morocco: the oldest record of repetitive shell morphologies among some seep bivalve molluscs. J. Syst. Palaeontol..

[13] Campbell, K. A. & Bottjer, D. J. *Peregrinella*: An early cretaceous cold-seep-restricted brachiopod. *Paleobiology* **21** (1995).

[14] Peckmann J, Walliser OH, Riegel W, Reitner J (1999). Signatures of hydrocarbon venting in a Middle Devonian carbonate mound (Hollard Mound) at the Hamar Laghdad (Antiatlas, Morocco). Facies.

[15] Jakubowicz M, Dopieralska J, Belka Z (2015). Tracing the composition and origin of fluids at an ancient hydrocarbon seep (Hollard Mound, Middle Devonian, Morocco): A Nd, REE and stable isotope study. Geochim. Cosmochim. Ac..

[16] Barbieri R, Ori GG, Cavalazzi B (2004). A Silurian Cold-Seep Ecosystem From the Middle Atlas, Morocco. Palaios.

[17] Ager DV, Cossey SPJ, Mullin PR, Walley CD (1976). Brachiopod ecology in Mid-Palaeozoic sediments near Khenifra, Morocco. Palaeogeogr. Palaeocl..

[18] Buggisch W, Krumm S (2005). Palaeozoic cold seep carbonates from Europe and NorthAfrica—an integrated isotopic and geochemical approach. Facies.

[19] Peckmann J, Campbell KA, Walliser OH, Reitner J (2007). A Late Devonian hydrocarbon-seep deposit dominated by dimerelloid brachiopods, Morocco. Palaios.

[20] Cope, J. C. W. A new look at early bivalve phylogeny. *Geological Society, London, Special Publications*1**77**, 81–95 (2000).

[21] Peckmann J, Gischler E, Oschmann W, Reitner J (2001). An Early Carboniferous seep community and hydrocarbon-derived carbonates from the Harz Mountains, Germany. Geology.

[22] Little CTS, Maslennikov VV, Morris NJ, Gubanov AP (1999). Two Palaeozoic hydrothermal vent communities from the southern Ural Mountains, Russia. Palaeontology.

[23] Krylova EM, Sahling H, Janssen R (2010). *Abyssogena*: a new genus of the family *Vesicomyidae* (Bivalvia) from deep-water vents and seeps. J. Mollus. Stud..

[24] Kelly SRA, Blanc E, Price SP, Whitham AG (2000). Early Cretaceous giant bivalves from seep-related limestone mounds, Wollaston Forland, Northeast Greenland. Geological Society, London, Special Publications.

[25] Kiel S, Peckmann J (2008). Paleoecology and Evolutionary Significance Of An Early Cretaceous Peregrinella-Dominated Hydrocarbon-Seep Deposit On The Crimean Peninsula. Palaios.

[26] Samtleben C, Munnecke A, Bickert T (2000). Development of Facies and C/O-Isotopes in Transects through the Ludlow of Gotland: Evidence for Global and Local Influences on a Shallow-marineEnvironment. Facies.

[27] Lehnert O (2007). δ^13^C records across the late Silurian Lau event: New data from middle palaeo-latitudes of northern peri-Gondwana (Prague Basin, Czech Republic). Palaeogeogr. Palaeocl..

[28] Peckmann J, Kiel S, Sandy MR, Taylor DG, Goedert JL (2011). Mass Occurrences of the Brachiopod *Halorella* in Late Triassic Methane-Seep Deposits, Eastern Oregon. J.Geol..

[29] Peckmann J, Sandy MR, Taylor DG, Gier S, Bach W (2013). An Early Jurassic brachiopod-dominated seep deposit enclosed by serpentinite, eastern Oregon, USA. Palaeogeogr. Palaeocl..

[30] Kiel S (2014). The Paleoecology, Habitats, and Stratigraphic Range of the Enigmatic Cretaceous Brachiopod *Peregrinella*. PLoS ONE.

[31] Kiel S, Krystyn L, Demirtaş F, Koşun E, Peckmann J (2017). Late Triassic mollusk-dominated hydrocarbon-seep deposits from Turkey. Geology.

[32] Peckmann J, Birgel D, Kiel S (2009). Molecular fossils reveal fluid composition and flow intensity at a Cretaceous seep. Geology.

[33] Kaim A, Bitner MA, Jenkins RG, Hikida Y (2010). A monospecific assemblage of terebratulide brachiopods in the Upper Cretaceous seep deposits of Omagari, Hokkaido, Japan. Acta Palaeontol. Pol..

[34] Sandy, M. R. in The Vent and Seep Biota: Aspects from Microbes to Ecosystems Vol. 33 Topics in Geobiology (ed S. Kiel) 279–314 (Springer, 2010).

[35] James, M. A. *et al*. in *Advances in Marine Biology* Vol. Volume 28 (eds J. H. S. Blaxter & A. J. Southward) 175–387 (Academic Press, 1992).

[36] Rhodes MC, Thompson RJ (1993). Comparative physiology of suspension-feeding in living brachiopods and bivalves - evolutionary implications. Paleobiology.

[37] Pin C, Briot D, Bassin C, Poitrasson F (1994). Concomitant separation of strontium and samarium-neodymium for isotopic analysis in silicate samples, based on specific extraction chromatography. Anal. Chim. Acta.

[38] Dopieralska, J. *Neodymium isotopic composition of conodonts as a palaeoceanographic proxy in the Variscan oceanic system* Ph.D. thesis, Justus-Liebig-University, (2003).

[39] Balinski A, Biernat G (2003). New observations on rhynchonelloid brachiopod *Dzieduszyckia* from the Famennian of Morocco. Acta Palaeontol. Pol..

[40] Sandy MR, Hryniewicz K, Hammer O, Nakrem HA, Little CT (2014). Brachiopods from Late Jurassic-Early Cretaceous hydrocarbon seep deposits, central Spitsbergen, Svalbard. Zootaxa.

