# Supplementary material for: Mass occurrence of seep-specific bivalves in the oldest-known cold seep metazoan community
Source: Sci Rep. 2017 Oct 30;7:14292. doi: 10.1038/s41598-017-14732-y (PMC5662593; doi:10.1038/s41598-017-14732-y)
Supplement: Supplementary file 1 — Supplementary Information [file 41598_2017_14732_MOESM1_ESM.pdf]

Mass occurrence of seep-specific bivalves in the oldest-known cold seep metazoan community

Michał Jakubowicz, Krzysztof Hryniewicz, Zdzislaw Belka

Supplementary discussion

Evaluation of previous explanations for the ^{13}C-enrichment of the seep carbonates

The composition of the fluids emitted at the El Borj seep, and thus the environmental background of the studied fossil community remained for a long time a matter of controversy. Barbieri et al. (2004) originally interpreted the few positive carbon isotope values measured by them as diagenetic signals that must have overprinted the originally more negative values in the course of diagenesis. Such a perception is, however, difficult to accept. While diagenetic alteration may, indeed, result in shifting δ^{18}O ratios towards lower signatures, conspicuous change in the δ^{13}C values requires much higher fluid-rock ratios, which are rarely attainable during burial in the marine realm (Banner and Hanson, 1990; Marshall, 1992; Brand et al., 2011; see also Jakubowicz et al., 2015). If one assumes the El Borj carbonates to represent typical seep limestones, such a scenario would entail a dramatic diagenetic shift from strongly negative to strongly positive δ^{13}C values, and hence comprising at least several, and possibly a few tens of ‰. No examples of similarly drastic diagenetic exchange have been reported from any other seep carbonates to date. Furthermore, due to progressive degradation of organic matter, diagenetic fluids are most commonly typified by negative δ^{13}C values, and thus cannot account for the appearance of abnormally heavy carbon isotope ratios of carbonates. Sure enough, the blocky calcite crystals that close the studied paragenetic sequence reveal significantly ^{13}C-depleted isotope signatures. Finally, diagenetic alteration results typically in the wide scatter of the observed values, ranging from strongly altered to relatively pristine ones (e.g., Patterson & Walter, 1994; Brand et al., 2004; Knauth & Kennedy, 2009), and, indeed, such trends are visible for the oxygen and strontium isotope ratios (Supplementary Fig. 4). Notably, while for the early isopachous cements the oxygen isotope values show moderate ^{18}O-depletion relative to the signal of contemporaneous seawater, the strontium isotope signatures, another geochemical proxy considerably more susceptible to diagenetic overprinting than carbon isotopes (Banner and Hanson, 1990; Banner, 1995; Jaffrés et al., 2007), fall within or close to the range typical of upper Silurian marine calcites.

The only process that may be potentially responsible for supplying locally significant quantities of isotopically heavy carbon during early marine diagenesis is methanogenesis, and this process was specifically invoked by Buggisch and Krumm (2005) and Himmler et al. (2008) to account for the positive δ^{13}C values of the El Borj carbonates. The dominant role of ^{13}C-rich, residual CO_2 produced by methanogenesis in the development of the observed signals is, however, unlikely. Methanogenesis, as a microbe-driven process associated with little free energy change, operates at depth in the sediment column and rarely results in the formation of more extensive carbonate beds. Instead, diagenesis in the zone of methane production is most often recorded in seep carbonates, if at all, by few ^{13}C-enriched cement generations infilling pore spaces available in otherwise ^{13}C-depleted limestones that form due to oxidation of the produced methane (e.g., Sample & Reid, 1998; Naehr et al., 2007; Kuechler et al., 2012). In fact, until methane reaches the zone where the inflow of seawater-derived sulphate enables anaerobic methane oxidation, CO_2 produced

by methanogenesis will result in decrease, rather than increase in pH, and hence will not promote voluminous carbonate precipitation. In rare instances where methanogenesis-associated, isotopically heavy carbonate bodies have been identified, they are invariably dominated by dolomite, and reveal no fibrous or botryoidal cement fabrics indicative of increased fluid flow, nor do they engulf remains of seep-related, oxygen-dependent metazoans (Greinert et al., 2001; Stakes et al., 1999, 2002; Orphan et al., 2004; Naehr et al., 2007; Paull et al., 2007). Therefore, we consider both the diagenetic, as well as methanogenesis-related origin of the observed anomalous ^{13}C-enrichment of the El Borj seep carbonates unlikely.

Stratigraphic position of the seep deposit and its implications for interpretation of the isotope data

Establishing precise stratigraphy of the seep carbonate body at El Borj poses some difficulties. As is commonly the case for authigenic carbonates that form at seeps, none of the several samples of the El Borj limestones analysed by us yielded conodonts. In addition, direct contact between the seep carbonates and surrounding sediments cannot be observed. Based on the presence of the crinoid *Scyphocrinus elegans* found in the surrounding deposits, Ager et al. (1976) tentatively assigned the El Borj limestones to the upper Silurian. Subsequently, a single sample containing the Pridolian (uppermost Silurian) conodont *Ozarcodina remscheidensis eosteinhornensis* was reported by Buggisch and Krumm (2005), but the authors provided no details as to the position of the sample within the seep carbonate body or surrounding sediments.

All previous workers assumed the seep deposit to be preserved in place within contemporaneous deposits. Ager et al. (1976) wrote that 'The limestone lens appears to be emplaced within a dominantly shaly sequence with thin crinoidal limestones and ripple-laminated sandstones, though no direct contacts could be seen. Scattered around on the ground hereabouts, however, and evidently derived from the shale are abundant crinoids and orthocone nautiloids with sponges, gastropods, bivalves and solitary corals found nearby in the same horizons (Cossey, 1974). The crinoids, which often consist of many still articulated columnals, calycal plates and brachials, are clearly attributable to the species *Scyphocrinus elegans* Zenker of which a complete calyx was found." Barbieri and co-workers (2004) shortly state that 'The limestone is embedded in an informally named, upper Silurian siliciclastic sequence ("Série à faciés flysch" of Willefert, 1963) in the Meseta domain of the Middle Atlas (Michard, 1976), and a similar description is given by Buggisch and Krumm (2005): 'The lenticular carbonate body is embedded in a shaly sequence ("Série à faciés flysch" of Willefert 1963) of the Moroccan Meseta. Ager et al. (1976) assigned a Late Silurian to Early Devonian age to the mound based on the occurrence of the echinoderm *Scyphocrinus elegans* Zenker and the atrypid brachiopod *Dubaria lantenoisi* Termier.'

Our conodont data show that the shaly deposits represent the *crispa* Zone of the upper Ludfordian (upper Silurian). A thin, micritic limestone bed, located about 0.8 m below the Unit A of the El Borj limestones, has produced a rich conodont fauna with *Ozarkodina confluens*, *Zieglerodina*? *zellmeri*, and *Ozarkodina remscheidensis*. Another sample of crinoid-bearing micritic limestone collected from a pocket within the seep carbonate of the Unit C (Supplementary Figure 1j) has yielded *Ozarkodina crispa* and some few indeterminate ramiform elements. The precise stratigraphic range of *O. crispa* has been recently subjected to some debates; this species has been traditionally regarded as indicative of the latest Ludfordian, but there are indications that locally it might have appeared somewhat earlier (Loydell and Fryda, 2011; Kaljo et al., 2015). Regardless of these minor interpretational differences as to the first appearance of *O. crispa* within the upper

Ludfordian, the presence of the species corresponds to the final stages of the very prominent positive excursion in the carbon isotope composition of seawater succeeding the mid-Ludfordian Lau Event, a ~1 Myr-long interval during which $\delta^{13}C$ signals of marine calcites displayed exceptionally high values ranging from +6 up to even +11 (e.g., Samtleben et al., 2000; Calner et al., 2005; Lehnert et al., 2007; Loydell and Fryda, 2011; Jarochowska and Kozłowski, 2014). Therefore, we suggest that the apparent contradiction between the petrological and palaeoecological features of the El Borj carbonates, and their isotopic signals is a consequence of unusual high $\delta^{13}C$ signatures of the ambient seawater, rather than an anomalous effect of diagenetic processes. Hence, the seep carbonates were, in fact, depleted in ^{13}C relative to the signature of seawater, as is typical of seep precipitates.

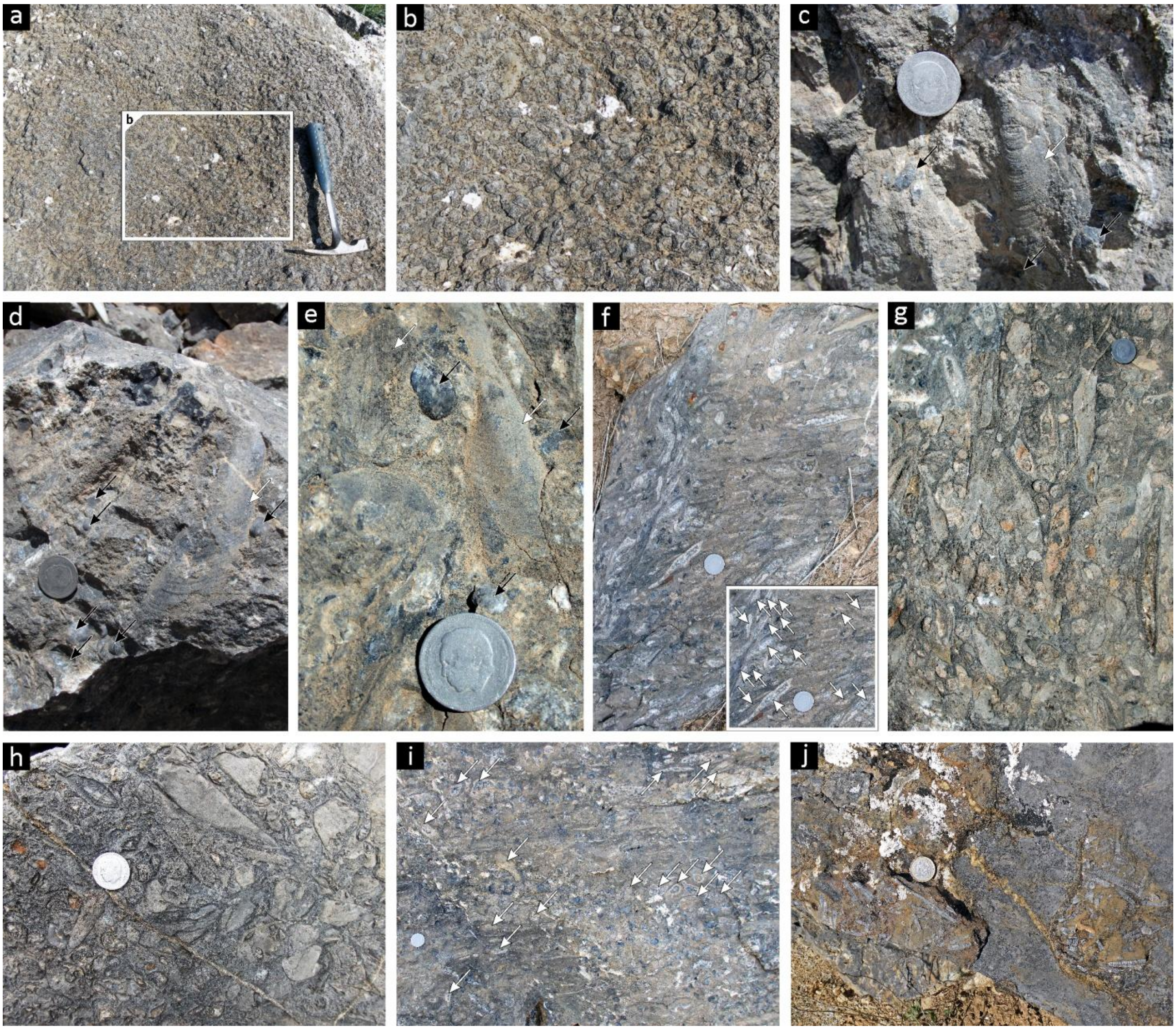

Supplementary Figure 1. Upper Silurian hydrocarbon seep carbonates of El Borj (Moroccan Meseta) observed in the field. a,b, The micritic carbonates with mass concentrations of the atrypid brachiopod *Septatrypa lantenoisi* characteristic of the lower, stratigraphically older portion of the seep carbonate body (Unit B *sensu* Barbieri et al., 2004; see Fig. 1a). Hammer for scale. **c–i**, Fibrous-cement dominated carbonates hosting tight aggregates of *S. lantenoisi* and the large modiomorphid bivalve *Ataviaconcha* sp. (Unit C *sensu* Barbieri et al., 2004; see Fig. 1a). **c–e**, relatively well preserved specimens of *Ataviaconcha* sp. (white arrows) exposed on the surface of the seep limestones. Note the abundant presence of brachiopods (black arrows). **f–i**, Typical exposures of the studied bivalve-brachiopod assemblage. As a guide for identification of the bivalves in the field view, the bivalve shells seen in the central part of **f** are indicated with arrows in the inset (see also Fig. 2f,g). While the brachiopod shells are relatively well preserved and easy to identify, the bivalves commonly show irregular outlines due to recrystallisation or dissolution of their shells. As a consequence, in some portions of the deposit recognising the original shell outlines may pose difficulties (**i**), which can possibly be held responsible for the abundant presence of the bivalves being unnoticed in previous studies (Ager et al., 1976; Barbieri et al., 2004; Buggisch & Krumm, 2005). In fact, Barbieri et al. (2004) figured a thin section-view of a few bivalve specimens in their Fig. 9b, whereas Buggisch & Krumm (2005) refer to the presence of 'very abundant irregular, spar-filled cavities', some of which may have represented the cement-filled shell interiors that comprise the majority of large open spaces available for cementation in the unit C (arrowed in **i**). Coin (21 mm in diameter) for scale. **j**, Pockets of crinoid-rich carbonate sediment found within the seep limestones. This facies contains the upper Ludfordian conodont *Ozarcodina crispa*. Coin (23 mm in diameter) for scale.

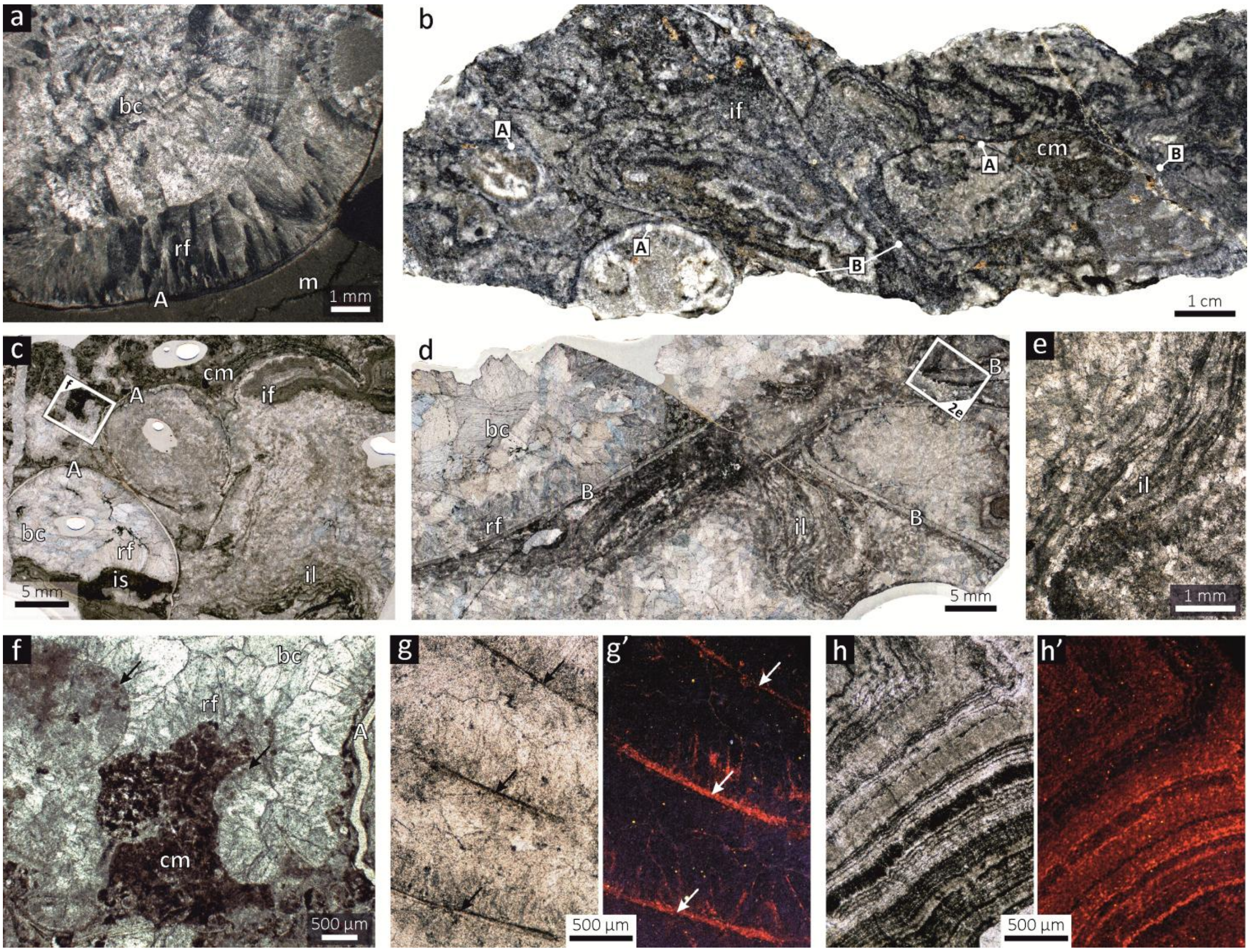

Supplementary Figure 2. Petrological features of the hydrocarbon seep carbonates of El Borj. a, Photomicrograph (cross-polarised light) showing the two main cement generations infilling the brachiopod shells found in the seep carbonates of the Unit B (*sensu* Barbieri et al., 2004; see Fig. 1a). The radiaxial-fibrous cement indicative of increased fluid flow is not universally present, with some of the shells filled solely by bladed-to-blocky spar characteristic of relatively slow cementation under low-energy conditions. **b–h**, Carbonates embedding the bivalve-brachiopod assemblage of the Unit C (*sensu* Barbieri et al., 2004; see Fig. 1a). **b**, Polished slab of the seep limestone with visible sections of bivalve and brachiopod shells. Note the intricate appearance of the carbonates, reflecting a very complex paragenetic sequence typical of seep precipitates and resulting from strong spatial and temporal variability in the conditions of the cement growth. **c–f**, Thin-section views (plane-polarised light) of the seep carbonates showing their irregular, spatially heterogenous fabrics with abundance of microbial-derived (clotted, laminated) fabrics and important role of fibrous and radiaxial-fibrous cements, a large proportion of which were diagenetically altered to a mosaic of fine spar crystals. Other than the bivalves and brachiopods, the fossils are rare and include mostly small, poorly preserved gastropods (**f**). **g–g'**, Photomicrographs showing transmitted, plane-polarised light (**g**) and cathodoluminescence (**g'**) views of the cements encrusting the brachiopod brachidium. The brachidium gives conspicuous, orange luminescence response indicative of some degree of its alteration during early burial. Despite its partial recrystallisation, the fibrous cement that grew on the skeletal elements has mostly retained its primary lack of luminescence indicative of precipitation under near-surface, oxygen-rich conditions. **h–h'**, Photomicrographs showing transmitted, plane-polarised light **(h)** and cathodoluminescence (**h'**) views of the isopachous, originally fibrous cement. Different growth laminae are defined by both variations in the amount of impurities and crystal lattice defects (transmitted-light), and different luminescence responses (cathodoluminescence). The red-to-orange luminescence pattern of the cement attests some degree of elemental exchange that accompanied its partial diagenetic recrystallisation. *Abbreviations*: carbonate phases making up the seep carbonates: m – micrite; cm – clotted micropeloidal micrite; is – internal sediment; rf – radiaxial-fibrous cement; if – isopachous fibrous cement; il – microstromatolitic, isopachous laminated cement; bc – blocky calcite spar; fossils: B – modiomorphid bivalves; A – atrypid brachiopods.

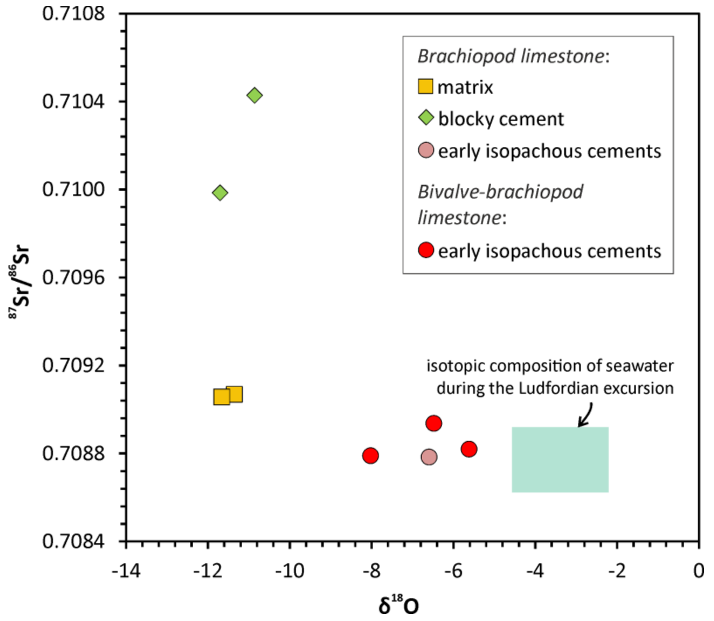

Supplementary Figure 3. Strontium vs. oxygen isotope cross-plot for various carbonate phases found in the carbonates hosting the monospecific brachiopod accumulation (Unit B) and the bivalve-brachiopod assemblage (Unit C; the units after Barbieri et al., 2004, see text). The range of the isotopic composition of contemporaneous, late Ludfordian ambient seawater (Azmy et al., 1999; Samtleben et al., 2000) is shown for comparison. Error bars are of the same size as or shorter than the symbols. The $\delta^{18}O$ values are shown in ‰ V-PDB.

Supplementary Table 1. Carbon, oxygen and strontium isotope signatures of the El Borj seep carbonates.

Carbonate phase	$\delta^{13}C$ (‰ V-PDB)	$\delta^{18}O$ (‰ V-PDB)	$^{87}Sr/^{86}Sr$
brachiopod limestone			
matrix	0.16	-11.66	0.709057±30
matrix	1.72	-11.36	0.709069±17
matrix	0.14	-10.03	
matrix	-0.89	-11.30	
internal sediment	-0.98	-10.98	
internal sediment	0.50	-8.64	
isopachous cement	2.85	-9.35	
isopachous cement	0.95	-6.60	0.708784±9
isopachous cement	3.90	-6.54	
isopachous cement	-2.25	-6.45	
isopachous cement	1.19	-7.36	
blocky cement	-0.64	-10.52	
blocky cement	-5.65	-11.70	0.709985±15
blocky cement	-0.53	-10.86	0.710428±11
blocky cement	-4.13	-11.10	
blocky cement	-2.44	-12.33	
bivalve-brachiopod limestone			
isopachous cement	0.38	-5.70	
isopachous cement	0.52	-5.41	
isopachous cement	2.59	-4.49	
isopachous cement	3.13	-5.36	
isopachous cement	3.15	-5.43	
isopachous cement	5.02	-4.54	
isopachous cement	2.99	-4.58	
isopachous cement	-2.38	-8.02	0.70879±14
isopachous cement	1.21	-7.71	
isopachous cement	1.57	-4.16	
isopachous cement	0.51	-7.92	
isopachous cement	-2.43	-5.78	
isopachous cement	1.21	-5.50	
isopachous cement	4.00	-6.54	
isopachous cement	4.75	-7.20	
isopachous cement	1.28	-6.48	0.708937±29
isopachous cement	-1.71	-5.62	0.70882±14
isopachous cement	-2.80	-5.30	
isopachous cement	-1.93	-7.96	
isopachous cement	2.32	-5.32	
isopachous cement	7.18	-6.17	
isopachous cement	0.12	-7.87	
isopachous cement			0.709080±10
blocky cement	1.20	-5.13	
blocky cement	0.97	-5.44	
blocky cement	-2.44	-11.57	
blocky cement	-4.07	-11.98	
blocky cement	0.95	-10.08	
blocky cement	-4.40	-10.37	

References

Ager DV, Cossey SPJ, Mullin PR, Walley CD, 1976. Brachiopod ecology in Mid-Palaeozoic sediments near Khenifra, Morocco. *Palaeogeography Palaeoclimatology Palaeoecology* 1976, **20:** 171-185.

Balinski A, Biernat G. New observations on rhynchonelloid brachiopod *Dzieduszyckia* from the Famennian of Morocco. *Acta Palaeontologica Polonica* 2003, **48**(3)**:** 463-474.

Banner JL, Hanson GN. Calculation of simultaneous isotopic and trace-element variations during water-rock interactions with applications to carbonate diagenesis. *Geochimica Et Cosmochimica Acta* 1990, **54**(11)**:** 3123-3137.

Banner JL. Application of the trace element and isotope geochemistry of strontium to studies of carbonate diagenesis. *Sedimentology* 1995, **42:** 805-824.

Barbieri R, Ori GG, Cavalazzi B. A Silurian Cold-Seep Ecosystem From the Middle Atlas, Morocco. *Palaios* 2004, **19:** 527–542.

Brand U. Carbon, oxygen and strontium isotopes in Paleozoic carbonate components: an evaluation of original seawater-chemistry proxies. *Chemical Geology* 2004, **204**(1-2)**:** 23-44.

Brand U, Logan A, Bitner MA, Griesshaber E, Azmy K, Buhl D. What is the ideal proxy for Palaeozoic seawater chemistry? *Memoirs of the Association of Australasian Palaeontologists* 2011, **41:** 9-24.

Buggisch W, Krumm S. Palaeozoic cold seep carbonates from Europe and North Africa—an integrated isotopic and geochemical approach. *Facies* 2005, **51**(1-4)**:** 566-583.

Calner M. A Late Silurian extinction event and anachronistic period. *Geology* 2005, **33**(4): 305.

Gischler E, Sandy MR, Peckmann J. *Ibergirhynchia contraria* (F. A. Roemer, 1850), an Early Carboniferous seep-related rhynchonellide brachiopod from the Harz Mountains, Germany - A possible successor to *Dzieduszyckia*? *Journal of Paleontology* 2003, **77**(2)**:** 293-303.

Greinert J, Bohrmann G, Suess E. Gas Hydrate-Associated Carbonates and Methane-Venting at Hydrate Ridge: Classification, Distribution, and Origin of Authigenic Lithologies. In: Paull CK, Dillon PW (eds). *Natural Gas Hydrates: Occurrence, Distribution, and Detection*, 2001, pp 99-113.

Himmler T, Freiwald A, Stollhofen H, Peckmann J. Late Carboniferous hydrocarbon-seep carbonates from the glaciomarine Dwyka Group, southern Namibia. *Palaeogeography, Palaeoclimatology, Palaeoecology* 2008, **257**(1-2)**:** 185-197.

Hryniewicz K, Jakubowicz M, Belka Z, Dopieralska J, Kaim A. New bivalves from a Middle Devonian methane seep in Morocco: the oldest record of repetitive shell morphologies among some seep bivalve molluscs. *Journal of Systematic Palaeontology* 2017, **15**(1)**:** 19-41.

Jaffrés JBD, Shields GA, Wallmann K. The oxygen isotope evolution of seawater: A critical review of a long-standing controversy and an improved geological water cycle model for the past 3.4 billion years. *Earth-Science Reviews* 2007, **83**(1-2)**:** 83-122.

Jakubowicz M, Berkowski B, Lopez Correa M, Jarochowska E, Joachimski M, Belka Z. Stable Isotope Signatures of Middle Palaeozoic Ahermatypic Rugose Corals - Deciphering Secondary Alteration, Vital Fractionation Effects, and Palaeoecological Implications. *PLoS One* 2015, **10**(9)**:** e0136289.

Jarochowska E, Kozłowski W. Facies development and sequence stratigraphy of the Ludfordian (Upper Silurian) deposits in the Zbruch River Valley, Podolia, western Ukraine: local facies overprint on the δ13Ccarb record of a global stable carbon isotope excursion. *Facies* 2014, **60**(1): 347-369.

Jenkins R, Kaim A, Little C, Iba Y, Tanabe K, Campbell K. Worldwide distribution of the modiomorphid bivalve genus *Caspiconcha* in late Mesozoic hydrocarbon seeps. *Acta Palaeontologica Polonica* 2013, **58**(2)**:** 357-382.

Kaim A, Bitner MA, Jenkins RG, Hikida Y. A monospecific assemblage of terebratulide brachiopods in the Upper Cretaceous seep deposits of Omagari, Hokkaido, Japan. *Acta Palaeontologica Polonica* 2010, **55**(1)**:** 73-84.

Kaljo D, Einasto R, Martmaa T, Märss T, Nestor V, Viira V. A bio-chemostratigraphical test of the synchroneity of biozones in the upper Silurian of Estonia and Latvia with some implications for practical stratigraphy. *Estonian Journal of Earth Sciences* 2015, **64**(4): 267-283.

Knauth LP, Kennedy MJ. The late Precambrian greening of the Earth. *Nature* 2009, **460**(7256)**:** 728-732.

Kuechler RR, Birgel D, Kiel S, Freiwald A, Goedert JL, Thiel V, *et al.* Miocene methane-derived carbonates from southwestern Washington, USA and a model for silicification at seeps. *Lethaia* 2012, **45**(2)**:** 259-273.

Lehnert O, Frýda J, Buggisch W, Munnecke A, Nützel A, Křiž J, *et al.* δ^{13}C records across the late Silurian Lau event: New data from middle palaeo-latitudes of northern peri-Gondwana (Prague Basin, Czech Republic). *Palaeogeography, Palaeoclimatology, Palaeoecology* 2007, **245**(1-2)**:** 227-244.

Loydell DK, Frýda J. At what stratigraphical level is the mid Ludfordian (Ludlow, Silurian) positive carbon isotope excursion in the type Ludlow area, Shropshire, England? *Bulletin of Geosciences* 2011, **86**(2): 197-208.

Manceñido MO, Owen EF, Savage NM, Dagys AS. Dimerelloidea. In: Kaesler RL (ed). *Treatise on invertebrate paleontology, Part H, Brachiopoda revised*, vol. 4. Geological Society of America: Boulder, 2002, pp 1236–1245.

Marshall JD. Climatic and oceanographic isotopic signals from the carbonate rock record and their preservation. *Geological Magazine* 1992, **129**(2)**:** 143-160.

Naehr TH, Eichhubl P, Orphan VJ, Hovland M, Paull CK, Ussler W, *et al.* Authigenic carbonate formation at hydrocarbon seeps in continental margin sediments: A comparative study. *Deep Sea Research Part II: Topical Studies in Oceanography* 2007, **54**(11-13)**:** 1268-1291.

Orphan VJ, Ussler W, Naehr TH, House CH, Hinrichs KU, Paull CK. Geological, geochemical, and microbiological heterogeneity of the seafloor around methane vents in the Eel River Basin, offshore California. *Chemical Geology* 2004, **205**(3-4)**:** 265-289.

Patterson WP, Walter LM. Syndepositional diagenesis of modern platform carbonates: Evidence from isotopic and minor element data. *Geology* 1994, **22**(2)**:** 127-130.

Paull CK, Ussler W, Peltzer ET, Brewer PG, Keaten R, Mitts PJ, *et al.* Authigenic carbon entombed in methane-soaked sediments from the northeastern transform margin of the Guaymas Basin, Gulf of California. *Deep Sea Research Part II: Topical Studies in Oceanography* 2007, **54**(11-13)**:** 1240-1267.

Peckmann J, Kiel S, Sandy MR, Taylor DG, Goedert JL. Mass Occurrences of the Brachiopod *Halorella* in Late Triassic Methane-Seep Deposits, Eastern Oregon. *The Journal of Geology* 2011, **119**(2)**:** 207-220.

Sample JC, Reid MR. Contrasting hydrogeologic regimes along strike-slip and thrust faults in the Oregon convergent margin: Evidence from the chemistry of syntectonic carbonate cements and veins. *Geological Society of America Bulletin* 1998, **110**(1)**:** 48-59.

Samtleben C, Munnecke A, Bickert T. Development of Facies and C/O-Isotopes in Transects through the Ludlow of Gotland: Evidence for Global and Local Influences on a Shallow-marine Environment. *Facies* 2000, **43:** 1-38.

Sandy MR. Brachiopods from Ancient Hydrocarbon Seeps and Hydrothermal Vents. In: Kiel S (ed). *The Vent and Seep Biota*. Springer Science and Business Media, 2010, pp 279-314.

Schreiber HA, Bitner MA, Carlson SJ. Morphological Analysis of Phylogenetic Relationships Among Extant Rhynchonellide Brachiopods. *Journal of Paleontology* 2013, **87**(4)**:** 550-569.

Stakes DS, Orange D, Paduan JB, Salamy KA, Maher N. Cold-seeps and authigenic carbonate formation in Monterey Bay, California. *Marine Geology* 1999, **159**(1-4)**:** 93-109.

Stakes DS, Trehu AM, Goffredi SK, Naehr TH, Duncan RA. Mass wasting, methane venting, and biological communities on the Mendocino transform fault. *Geology* 2002, **30**(5)**:** 407.
